# Circulating MicroRNAs as Diagnostic Biomarkers for Motor Neuron Disease

**DOI:** 10.3389/fnins.2020.00354

**Published:** 2020-04-16

**Authors:** Lin Wang, Lijuan Zhang

**Affiliations:** ^1^Department of Emergency Medicine, Shengjing Hospital of China Medical University, Shenyang, China; ^2^Department of Obstetrics and Gynecology, Shengjing Hospital of China Medical University, Shenyang, China

**Keywords:** circulating microRNA, biomarker, motor neuron disease, neurodegeneration, blood, cerebral spinal fluid

## Abstract

Motor neuron disease (MND) is a kind of neurodegenerative disease that selectively invades spinal cord anterior horn cells, brainstem motor neurons, cortical pyramidal cells and the pyramidal tract. The main clinical features are the symptoms and signs of impaired upper and lower motor neurons, manifested as muscle weakness, atrophy and pyramidal tract signs. Histopathology has shown the disappearance of pyramidal cells in the motor cortex, loss of motor neurons in the anterior horn of the spinal cord and brainstem, and degeneration of the corticospinal tract. Due to the lack of effective treatment methods, the prognosis is generally poor, so it is of great significance to confirm the diagnosis early by various means. However, the current diagnosis of MND mainly relies on the combination of clinical manifestations and neurophysiological examinations, lacking effective means of early diagnosis. Circulating microRNA (CmiRNA) is a kind of stable miRNA molecule in serum, plasma and other body fluids, which has the characteristics of distinct differential expression, sensitive detection and convenient sample collection. As a possible new biomarker of MND, CmiRNA can not only reveal the pathophysiological process of MND, but also monitor disease progression and response to drug therapy. With the development of miRNA detection technology, more and more CmiRNAs as biomarkers with potential diagnostic value have been investigated. In this review, we explored the possibility of circulating samples as different sources of biomarkers for the diagnosis of MND, analyzing the progress of CmiRNA detection techniques, and presenting potential diagnostic MND biomarkers that have been reported.

## Introduction

Motor neuron disease (MND) is a kind of neurodegenerative disease that selectively invades spinal cord anterior horn cells, brainstem motor neurons, cortical pyramidal cells and the pyramidal tract, which was first reported by Charcot in 1869. The main clinical features are the symptoms and signs of impaired upper and lower motor neurons, manifested as muscle weakness, atrophy and pyramidal tract signs (Luo et al., 2019; Ragagnin et al., 2019). Histopathology has shown the disappearance of pyramidal cells in the motor cortex, loss of motor neurons in the anterior horn of the spinal cord and brainstem, and degeneration of the corticospinal tract (Statland et al., 2015; Taylor et al., 2016). At present, the pathogenesis of the disease is still unclear. Different theories have been put forward in research in biochemistry, molecular biology, cell biology and other basic medical fields (Bartoletti et al., 2019; Lai and Ichida, 2019; Yang Y. et al., 2019). The incidence and prevalence of MND vary with age, gender, race and region, and are related to genetic factors, occupational characteristics and special material contact history (Veldink, 2017; Tank et al., 2018). MND is mainly divided into four types: amyotrophic lateral sclerosis (ALS), progressive muscular atrophy (PMA), progressive bulbar palsy (PBP) and primary lateral sclerosis (PLS) (Statland et al., 2015; Goncalves et al., 2016). Due to the lack of effective treatment and poor prognosis of MND, it is of great significance to diagnose MND as early as possible by various means (Westeneng et al., 2018; Liu et al., 2019; Sharrad and Schultz, 2019). There is continuous research and development on various biomarkers of the disease. The levels of kynurenine pathway metabolites (KPMs) are known to be dysregulated in the serum, cerebrospinal fluid (CSF), and tissue of ALS patients (Chen et al., 2010), and Tan and Guillemin suggested that KPMs could be a potential sensitive and specific diagnostic biomarker for ALS patients (Tan and Guillemin, 2019). Neurofilaments (Nfs) are neuron-specific cytoskeletal proteins, and it was reported that increased levels in biological fluids are proportionally associated with degree of axonal damage, representing potential biomarkers in MND (Gaiottino et al., 2013). Gagliardi et al. (2019) believed that Nfs may be a promising biomarker for diagnosing MND, predicting disease progression, and reflecting response to pharmacological intervention. Pathological 43-kDa transactive responsive DNA-binding protein (TDP-43) has been validated as the major disease protein in ALS (Neumann et al., 2006). Geser et al. (2011) concluded that TDP-43 was also an MND/LMN or PMA proteinopathy similar to sporadic ALS, which might reflect MND progression. However, limited by low sensitivity and specificity, these markers are not suitable as a routine test for clinical examination.

Circulating miRNAs (CmiRNAs) are a class of miRNAs found in body fluids, mainly found in blood, urine, saliva, tears, milk, or amniotic fluid, which are mainly produced by the efflux of miRNAs from tissue or cells into biofluids (Jin and Xing, 2017). CmiRNAs in peripheral biofluids have been extensively investigated as biomarkers for early diagnosis and monitoring disease progression, such as Alzheimer’s disease (AD) and Parkinson’s disease (PD) (Hajjari et al., 2017; Li et al., 2017; Katsu et al., 2019). miRNAs are small non-protein-encoding RNAs, usually between 19 and 25 bases in length, that can bind to RISC to regulate mRNA expression. Dysregulated miRNAs have been reported associated with the pathogenesis of MND, permitting CmiRNAs to be biomarkers in biofluids for the diagnosis and progression of MND (De Paola et al., 2019; Katsu et al., 2019). This review summarizes the progress of research on the sources and functions of CmiRNAs, the potential of diagnostic markers, and the clinical application prospects, together with problems in MND-associated miRNA biomarkers.

## Source, Existence, Extraction, and Detection of miRNAs and Their Biomarker Potential

Under normal conditions, CmiRNAs are mainly derived from the active secretion of cells, the release of cells that are apoptotic or necrotic, and the lysis of circulating cells. In the disease state, some CmiRNAs may come from diseased tissue cells, such as tumor cells (Matamala et al., 2018). miRNAs may be passively leaked from a variety of damaged tissues or cells, like other substances. For example, miRNA-206 is specifically expressed in muscle tissue, and it can be detected in serum after involvement of muscle tissue in ALS patients (Valsecchi et al., 2015; Pegoraro et al., 2017). There are two forms of active secretion of miRNA: one is that the free miRNA is directly secreted by cells, and the other is that miRNA is first selectively wrapped in membrane structures such as exosomes and minute bodies, and then enters the humoral circulation from endocrinology to exocytosis in the wrapped form (Iguchi et al., 2010; Ogawa et al., 2010; Burgos et al., 2014; Srivastava et al., 2018).

The current methods of extracting miRNAs from biofluids are varied in different studies. Commonly used methods of miRNA extraction from biofluids include Trizol reagent and miRNA kits. Due to the small size and low expression of CmiRNAs fragments, quantitative detection is limited.

**FIGURE 1:**
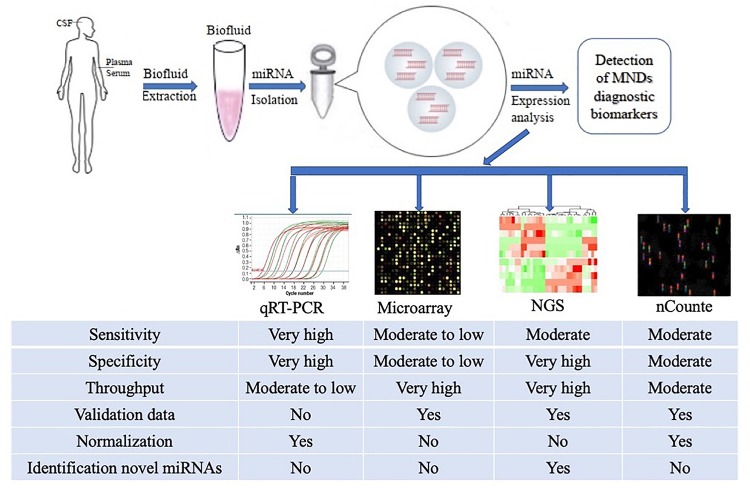
Methodological aspects to consider in the study of circulating miRNAs as biomarkers for ALS and other MNDs. The comparison among the common characteristics of miRNA detection platforms are summarized in the figure. qRT-PCR, quantitative Real-Time Polymerase Chain Reaction; NGS, Next Generation Sequencing.

Despite this, a number of effective CmiRNAs detection methods have been developed in recent years, including not only traditional cDNA library cloning, Northern blot and qRT-PCR, but also newly developed gene chip technology, high-throughput sequencing and nanostring nCounter (Morozova and Marra, 2008; Roberts et al., 2014; Ricci et al., 2018). With the development of molecular biology technology, the detection methods for CmiRNAs have been improved and developed, the new technologies enable the detection of CmiRNAs with greater sensitivity and precision, providing technical support for CmiRNAs to become diagnostic biomarkers (Figure 1).

CmiRNAs are not present in free form but are bound to specific proteins or encapsulated in active vesicles in the form of complexes in biofluids, thus providing the ability to resist RNase degradation and stable storage (Vickers et al., 2011; Zetterberg and Burnham, 2019). CmiRNAs have been repeatedly demonstrated to show substantial expression differences under different pathological conditions, which are closely related to the pathological state of individuals. Yang Q. et al. (2019) suggested that the serum level of miRNA-133b was significantly downregulated in AD patients, and that it may serve as a novel diagnostic biomarker for AD. miRNA-30c-5p has been reported to be significantly upregulated in multiple system atrophy (MSA) patients, and Vallelunga et al. (2019) considered that serum miRNA-30-5p could be a biomarker for the diagnosis and progression of MSA. Regarding CmiRNAs as diagnostic markers for MND diagnosis, relatively few studies have been published, but some breakthroughs have been made. In the next section, we review the findings of the most significant studies investigating the underlying role of CmiRNAs as diagnostic biomarkers in MND (Table 1).

**TABLE 1:** Aberrant expression of miRNAs as biomarkers in MND.

**Sample**	**MND**	**Sample size**	**Validated changes**		**RNA extraction**	**RT-qPCR validation**	**RT-qPCR normalization**	**References**
			**Up-regulated**	**Down-regulated**				
Serum	ALS	23 P, 30 C	miRNA-1, miRNA-19a-3p, miRNA-133a-3p, miRNA-133b, miRNA-192-3p, miRNA-192-5p, miRNA-144-5p	et-7d-3p, miRNA-139-5p, miRNA-320a, miRNA-320b, miRNA-320c, miRNA-425-5p	miRcury kit	SYBR- green-based RT-qPCR	miRNA-15b-5p, miRNA-19a-3p, miRNA-126-3p and miRNA-425-5p	Raheja et al., 2018
	sALS	20 P, 20 C	miRNA-142-3p	miRNA-1249-3p	Trizol LS	TaqMan miRNA RT-qPCR	miRNA-39	Matamala et al., 2018
	sALS	18 P, 16 C		miRNA-1234-3p, miRNA-1825	miRNeasy Mini kit	EXPRESS SYBR Green ER qPCR	miRNA-39-3p	Freischmidt et al., 2015
	sALS	23 P, 22 C	miRNA-143-3p, miRNA-206	miRNA-374b-5p	Circulating Nucleic Acid Isolation kit	miRNA TaqMan Low Density Arrays	miRNA-17-5p, miRNA-223-3p, miRNA-24	Waller et al., 2017b
Plasma	sALS	56 P, 20 C		let-7a-5p, let-7d-5p, let-7f-5p, let-7g-5p, let-7i-5p, miRNA-103a-3p, miRNA-106b-3p, miRNA-128-3p, miRNA-130a-3p, miRNA-130b-3p, miRNA-144-5p, miRNA-148a-3p, miRNA-148b-3p, miRNA-15a-5p, miRNA-15b-5p, miRNA-151a-5p, miRNA-151b, miRNA-16-5p, miRNA-182-5p, miRNA-183-5p, miRNA-186-5p, miRNA-22-3p, miRNA-221-3p, miRNA-223-3p, miRNA-23a-3p, miRNA-26a-5p, miRNA-26b-5p, miRNA-27b-3p, miRNA-28-3p, miRNA-30b-5p, miRNA-30c-5p, miRNA-342-3p, miRNA-425-5p, miRNA-451a, miRNA-532-5p, miRNA-550a-3p, miRNA-584-5p, miRNA-93-5p	PAXgene Blood RNA kit	TaqMan Advanced miRNA Cards	miRNA-331-3p, miRNA-423-3p, miRNA-423-5p, miRNA 484, miRNA-320a	Liguori et al., 2018
	sALS	16 P, 10 C	miRNA-4649-5p	miRNA-4299	LNA microRNA Array Power Labeling kit	Biosystems StepOnePlus RT-PCR	miRNA-4516	Takahashi et al., 2015
	ALS	5 P, 5 C	miRNA-4736, miRNA-4700-5p, miRNA-1207-5p, miRNA-4739, miRNA-4505, miRNA-24-3p, miRNA-149-3p, miRNA-4484, miRNA-4688, miRNA-4298, miRNA-939-5p, miRNA-371a-5p, miRNA-3619-3p	miRNA-1268a, miRNA-2861, miRNA-4508, miRNA-4507, miRNA-3176, miRNA-4745-5p, miRNA-3911, miRNA-3605-5p, miRNA-150-3p, miRNA-3940-3p, miRNA-4646-5p, miRNA-4687-5p, miRNA-4788, miRNA-4674, miRNA-1913, miRNA-634, miRNA-3177-3p	3D-Gene miRNA labeling kit	The 3D-Gene Human miRNA Oligo Chip		Katsu et al., 2019
CSF	sALS	24 P, 24 C	miRNA-181a-5p	miRNA-15b-5p, miRNA-21-5p	miRNeasy Mini kit	miFinder 384HC miRNA PCR array	miRNA-39-3p	Benigni et al., 2016
	sALS	32 P, 16 C	miRNA-124-3p, miRNA-126-5p, miRNA-127-3p, miRNA-23b-3p, miRNA-27b-3p, miRNA-9-5p, miRNA-99b-5p	let-7f-5p, miRNA-i50-5p, miRNA-142-5p, miRNA-378a-3p	miRVana PARIS kit	CFX384 BioRad RT-PCR	miRNA-204-5p, miRNA-10a-5p, miRNA-10b-5p, miRNA-30a-5p	Waller et al., 2017b
PBMCs	ALS	5 P, 5 C	miRNA-183, miRNA-193b, miRNA-451, miRNA-3935		miRNeasy Mini kit	Bio-Rad CFX96 RT-PCR		Chen et al., 2016
PBMCs/serum/CSF	sALS	10 P, 10 C	miR-338-3p		Trizol LS	miRCURY LNA PCR system	let-7 in serum, miRNA-24 in CSF	De Felice et al., 2014
Serum	SMA	10 P, 7 C	miRNA-9, miRNA-132		miRNeasy Serum/Plasma kit	TaqMan small RNA Assay	cel-miRNA-39	Catapano et al., 2016

*CSF, cerebrospinal fluid; P, patients; C, controls; PBMCs, peripheral blood mononuclear leukocytes.*

## Circulating miRNAs as Biomarkers in ALS

Amyotrophic lateral sclerosis is a progressive paralytic disorder characterized by degenerative changes in brain and spinal motor neurons, usually with insidious local weakness and then rapid progression to the paralysis of most muscles, including the diaphragm (Van Zundert and Brown, 2017). According to the global ALS epidemiological survey, the number of new ALS patients per year is 1–2 per 100,000 people, and incidence and prevalence increase with age. About 10% of ALS cases are familial, and the rest are sporadic (Doi et al., 2014; Talbott et al., 2016). Abnormal protein aggregation is found in the cytoplasm of motor cells in most patients with ALS, mainly TAR DNA-binding protein 43 (TDP 43). Since the superoxide dismutase 1 (SOD1) gene was shown to be associated with ALS in 1993, at least 25 related genes have been identified to date, including TDP 43 and C9orf72 (Neumann et al., 2006; Therrien et al., 2016).

Serum levels of miRNA in ALS patients were analyzed in four studies by using either qRT-PCR or microarray. Raheja et al. enrolled 23 ALS patients (3 familial and 20 sporadic) and 30 healthy controls; they identified 13 differentially expressed miRNAs, including 7 upregulated (miRNA-1, miRNA-19a-3p, miRNA-133a-3p, miRNA-133b, miRNA-192-3p, miRNA-192-5p, and miRNA-144-5p) and 6 downregulated (let-7d-3p, miRNA-139-5p, miRNA-320a, miRNA-320b, miRNA-320c, and miRNA-425-5p) miRNAs in ALS patients compared to healthy controls using qRT-PCR. By logistic regression and ROC curve analysis, the 7 up-regulated miRNAs and 6 down-regulated miRNAs had ideal diagnostic value to distinguish between ALS and healthy controls (Raheja et al., 2018). In a study by Matamala et al. (2018) the differential expression of miRNAs was first detected in the serum of SOD1 transgenic mice by using next-generation sequencing. Further verification of differential miRNAs in ALS patients’ serum by qRT-PCR revealed that there was substantial dysregulated expression of miRNA-142-3p and miRNA-1249-3p. In addition, the authors found a negative correlation between miRNA-142-3p and revised ALS functional rating scale and suggested that the serum level of miRNA-142-3p could be a potential biomarker for ALS severity (Matamala et al., 2018). In a qRT-PCR analysis of 18 sporadic ALS (sALS) patients and 16 healthy controls, Freischmidt et al. (2015) observed 2 significantly downregulated serum miRNAs (miRNA-1234-3p and miRNA-1825), and demonstrated that there was dysregulation of miRNA-1825 in both sALS and familial ALS (fALS) patients, and that significant downregulation of miRNA-1234-3p was restricted to patients with sALS. Another study including a larger number of sALS patients in validation cohorts distinguished a set of 3 CmiRNAs (miRNA-143-3p, miRNA-206, and miRNA-374b-5p) in serum. The levels of miRNA-143-3p and miRNA-206 were significantly increased, while the levels of miRNA-374b-5p were significantly decreased, which was not affected by Riluzole treatment. In a further analysis of the clinical significance of three candidate CmiRNAs by subgroup, Waller et al. determined that miRNA-143-3p was associated with lower limb onset of ALS patients (Waller et al., 2017a).

A number of studies have investigated plasma levels of miRNAs using high-throughput next-generation sequencing. In a study of dysregulated miRNAs associated with sALS, which included 56 ALS patients and 20 healthy controls, Liguori et al. (2018) identified 38 downregulated miRNAs in sALS patients. The authors also observed that the expression levels of miRNA-130a-3p, miRNA-151b, and miRNA-221-3p were positively correlated with sALS progression. These authors further suggested that these CmiRNAs could serve not only as biomarkers for diagnosis but also for monitoring disease progression (Liguori et al., 2018). In another study comparing plasma miRNAs between 16 sALS patients and 10 healthy controls using microarray analysis, validated by qPCR, Takahashi et al. (2015) found two differentially expressed miRNAs (downregulated miRNA-4299 and upregulated miRNA-4649-5p), regardless of other clinical features, concluding that these two plasma CmiRNAs had the potential to be ALS diagnosis biomarkers. By using microarray, Katsu et al. (2019) identified 30 differentially expressed miRNAs in ALS plasma, including 13 upregulated and 17 downregulated miRNAs, and they further investigated the relationship between these miRNAs and ALS, and suggested that four miRNAs (miRNA-24-3p, miRNA-1268a, miRNA-3911, and miRNA-4646-5) were enriched in synaptic vesicle exocytosis and processes. Detection of these biomarkers helps get a better understanding of ALS pathophysiology and could be used for early diagnosis (Katsu et al., 2019).

There are few studies on differential miRNA expression in CSF samples from ALS patients. Using qRT-PCR, Benigni et al. (2016) identified eight significantly deregulated miRNAs in CSF samples from 24 ALS patients, where statistical analysis results showed that upregulated miRNA-181a-5p and downregulated miRNA-15b-5p and miRNA-21-5p had the highest diagnostic accuracy, confirming the application potential as ALS diagnostic biomarkers. Eleven dysregulated miRNAs were detected by qRT-PCR in the CSF of sALS patients associated with neural and glial activity: 7 miRNAs (miRNA-124-3p, miRNA-126-5p, miRNA-127-3p, miRNA-23b-3p, miRNA-27b-3p, miRNA-9-5p, and miRNA-99b-5p) were upregulated and 4 miRNAs (let-7f-5p, miRNA-150-5p, miRNA-142-5p, and miRNA-378a-3p) downregulated (Waller et al., 2017b).

miRNA expression in the leukocytes of sALS patients was also addressed in one study. By using microarray technology in leukocytes obtained from 5 sALS patients, Chen et al. identified 11 differentially expressed miRNAs, including 4 upregulated and 7 downregulated miRNAs. The results of four of seven miRNAs (miRNA-183, miRNA-193b, miRNA-451, and miRNA-3935) were validated by qRT-PCR, and the authors showed that all these four miRNAs showed high diagnostic accuracy for ALS (Chen et al., 2016).

miRNA-338-3p is a well-studied CmiRNA in the previous studies. De Felice et al. (2014) demonstrated that miRNA -338-3p was upregulated in peripheral leukocytes, serum, and CSF from sALS patients. Their results further showed signs of miRNA-338-3p being localized in the gray matter of spinal cord tissues from sALS patients, and the authors considered the miRNA to be an effective biomarker in the early diagnosis of sALS (De Felice et al., 2014).

In addition to microarray analysis and qRT PCR validation, most CmiRNA studies included the optimal cut-off value for ROC analysis (Takahashi et al., 2015; Liguori et al., 2018; Matamala et al., 2018; Raheja et al., 2018). In most studies, CmiRNAs have been used as biomarkers to distinguish ALS from healthy controls, while a few studies have used them to distinguish patients with fALS from those with sALS. No matter what the purpose of the studies, we found that most results suggested that CmiRNAs, as biomarkers, have high sensitivity (up to 94%) and specificity (up to 87%), indicating that CmiRNAs have great potential as biomarkers in clinical practice (Benigni et al., 2016; Verde et al., 2019).

Although the specimens are from different sources, it is not difficult to find that CmiRNAs have proven their clinical value in many aspects based on previous researches. miRNA-24-3p, miRNA-1268a, miRNA-3911, miRNA-4646-5, miRNA-4299, and miRNA-4649-5p in plasma could be potential CmiRNAs for ALS diagnosis, miRNA-181a-5p, miRNA-15b-5p, and miRNA-21-5p in CSF also had high diagnostic accuracy for ALS; miRNA-1234-3p, miRNA-143-3p, miRNA-206, and miRNA-374b-5p in serum were restricted to patients with sALS in distinguishing fALS. Serum miRNA-142-3p could be a potential biomarker for ALS severity; miRNA-130a-3p, miRNA-151b, and miRNA-221-3p in plasma were positively correlated with sALS progression. At the same time, we cannot deny that there are few repetitive CmiRNAs in previous studies, which may be related to the involvement of multiple genes associated with genetic ALS and have different pathological pathways related to RNA metabolism. All this reflects the complexity of ALS diagnosis, and future studies with larger patient numbers and a higher methodological standardization might promote the clinical application of CmiRNAs as diagnostic biomarkers.

## CmiRNAs as Biomarkers in Other MNDs

SMA is a single gene autosomal recessive genetic disease, which is mainly caused by homozygous deletion of *SMN1* gene. It is characterized by degeneration of spinal cord anterior horn cells and muscle atrophy and weakness (Lefebvre et al., 1995; Faravelli et al., 2015). Recent studies have confirmed a critical role of miRNAs in the pathogenesis of SMA. In addition, dysregulated motor neuron−specific miRNAs are not only involved in the SMA motor neuron phenotype but may also be biomarkers and therapeutic targets (Haramati et al., 2010; Kye and Goncalves Ido, 2014; Magri et al., 2018). It was reported that miRNA-146a was significantly upregulated in the SMA spinal cord mouse model, where Sison et al. (2017) investigated the upregulated miRNA-146a-induced motor neuron loss *in vitro* and suggested astrocyte-produced miRNA-146a may be a contributing factor in SMA pathology. Kye et al. (2014) observed an upregulated expression of miRNA-183 in SMA knockdown in cortical neurons and suggested that miRNA-183 regulates axonal local translation via the mTOR pathway. Catapano et al. (2016) examined the expression changes of miRNA-9, -132, and -206 in SMA mouse tissues and patient serum. The authors found that the expression of miRNA-9 and miRNA-132 was significantly decreased in the spinal cord of SMA mice, but the expression in muscle was significantly increased, and that the expression of miRNA-206 was increased both in spinal cord and muscle tissue. These three miRNAs were also differentially expressed in the serum of SMA mice, and this change was prior to that in spinal cord and muscle tissue. miRNAs were detected by qRT-PCR in the serum of SMA patients. The expression levels of miRNA-9 and miRNA-132 were significantly increased in the serum of patients, but there was no significant change in miRNA-206 levels, suggesting that miRNA-9 and miRNA-132 can serve as non-invasive biomarkers (Catapano et al., 2016). Despite limited current researches, motor neuron-specific miRNAs may have great potential in the diagnosis of SMA. For example, miRNA-9 and miRNA-132 in serum, miRNA-206 in muscle tissue could be used to diagnose SMA. However, more clinical studies are needed to further validate the diagnostic efficacy of these CmiRNAs.

## Advantages and Problems of CmiRNAs as Biomarkers

At present, biomarkers in biofluid are mainly based on the change in expression level of some specific proteins, such as β-amyloid (Aβ) and tau protein in peripheral blood, for diagnosis of AD (McDade and Bateman, 2017), and detection of α-synuclein for diagnosis of PD and monitoring disease progression as well (Atik et al., 2016). However, the complexity of protein composition, the variation caused by post-transcriptional modification, the low level of protein biomarkers, the stability of samples and the sensitivity of assays restrict the further search for new protein biomarkers to some extent.

In clinical practice, miRNAs have been identified in blood, urine, saliva and other easily available biofluids. These CmiRNAs show extraordinary stability and anti-degradation ability, showing the basic characteristics of miRNAs in biofluid as ideal for disease biomarkers (Mushtaq et al., 2016). First, some of the CmiRNAs are released into membrane-bound vesicles by the cells, which can protect them from RNase degradation. These CmiRNAs are transported by vesicles, which come from the release of multivesicular bodies and exosomes (Orozco and Lewis, 2010). Secondly, 90% of miRNAs in blood are in the form of a nucleic acid-protein complex, because most of the blood CmiRNAs are associated with argonaute2 (ago2) protein. Ago2 is an effective component of miRNA induced silent complex (miRISC). It can directly bind to miRNAs and mediate the post-transcriptional regulation of mRNA. The reason why they can exist outside the cell is the high stability of Ago2-miRNA protein complexes (Bose and Bhattacharyya, 2018). In addition, CmiRNAs can also bind to non-vesicular lipoproteins and RNA-binding proteins. Protein-binding miRNA is a signal molecule which is actively secreted by cells and functions in intercellular communication. Therefore, CmiRNAs analysis is mainly to detect the level of miRNA-protein complexes, rather than vesicles (Wang et al., 2010; Vickers et al., 2011).

However, there are still many problems to be solved for the use of CmiRNAs as disease biomarkers. (1) Current research specimens are small in quantity, and systematic large-scale collaborative studies are lacking; at the same time, due to differences in detection methods and assessment standards, some research results and conclusions lack consistency. (2) The low efficiency of extracting miRNAs and the limited sensitivity and specificity of CmiRNA detection, which limits the application of miRNAs as disease biomarkers in clinical practice. In addition, the expression levels of miRNAs may change in a variety of diseases, such as miRNA-9, which can be differentially expressed in Huntington’s disease, SMA and PD (Catapano et al., 2016; Chang et al., 2017). (3) Studies have also found that serum and plasma miRNA expression profiles are not completely consistent. The mechanism of production and transport of serum or plasma miRNAs is still unclear (Wang et al., 2012).

## Conclusion

Emerging studies have shown that CmiRNAs can serve as potential biomarkers for neurodegenerative diseases and can be used in clinical diagnosis and monitoring disease progression and response to treatment measures. On the basis of the review of previous studies on the use of CmiRNAs as biomarkers of MND, we found that although the candidate CmiRNAs have very low reproducibility, it is not difficult to see after statistical analysis of the data obtained that CmiRNAs as diagnostic biomarkers have very ideal sensitivity and specificity. In addition, CmiRNAs can be easily detected and differentially expressed in a variety of body fluids, and can thus be promising diagnostic biomarkers for MND. To further apply CmiRNAs as biomarkers in clinical practice as soon as possible, it is necessary to ensure strict standardized test methods, as well as research and validation cohorts from multiple centers, and require selected patients to prioritize genetic diagnosis to improve the accuracy of the diagnosis.

## Author Contributions

LW wrote the manuscript primarily. LW and LZ produced the figure. LZ contributed to the editing of this review. All authors read and approved the final manuscript.

## Conflict of Interest

The authors declare that the research was conducted in the absence of any commercial or financial relationships that could be construed as a potential conflict of interest.
